# Supplementary material for: A novel viral RNA detection method based on the combined use of trans-acting ribozymes and HCR-FRET analyses
Source: PLoS One. 2024 Sep 26;19(9):e0310171. doi: 10.1371/journal.pone.0310171 (PMC11426510; doi:10.1371/journal.pone.0310171)
Supplement: S1 Appendix — The system allows users to select a color (red, green, or blue) in external component RGB LED using the keypad. The selected color is then displayed on corresponding RGB LED with feedback provided through the buzzer and visual confirmation on the LEDs and LCD. The code includes several libraries such as I2C communication with devices (Wire.h), supports I2C communication for the keypad (Keypad_I2C.h), managing the keypad input (Keypad.h), and handling interactions with the I2C-connected LCD display (LiquidCrystal_I2C.h). (DOCX) [file pone.0310171.s001.docx]

#include <Wire.h>

#include <Keypad_I2C.h>

#include <Keypad.h>

#include <LiquidCrystal_I2C.h>

#include <Arduino.h>

// Defining Pins

#define BUZZER_PIN 6 // Pin for the active buzzer

#define LED_RED_PIN 2 // Pin for TIP122 transistor Red LED

#define LED_GREEN_PIN 4 // Pin for TIP122 transistor Green LED

#define LED_BLUE_PIN 5 // Pin for TIP122 transistor Blue LED

// Defining display

#define endereco 0x27 // Defining address I2C of display in channel 0x27

#define colunas 16 // Defining column number on LCD display

#define linhas 2 // Defining rows number on LCD display

#define button 2

#define debounceTimeout 100

LiquidCrystal_I2C lcd(endereco, colunas, linhas);//Defining LCD display in use

// Defining 3x4 matrix keyboard

#define enderecoTeclado 0x26 // Defining address I2C of 3x4 matrix keyboard in channel 0x26

const byte ROWS = 4; // Defining rows number on keyboard

const byte COLS = 3; // Defining column number on keyboard

// array of keys

char keys[ROWS][COLS] = {

{'1','2','3'},

{'4','5','6'},

{'7','8','9'},

{'*','0','#'}

};

// Defining pins of PCF8574 i/o ports

byte rowPins[ROWS] = {3, 2, 1, 4}; //connect to the row pin outs of the keypad

byte colPins[COLS] = {5, 6, 7}; //connect to the column pin outs of the keypad

// Make objet to keyboard

Keypad_I2C keypad(makeKeymap(keys), rowPins, colPins, ROWS, COLS, enderecoTeclado);

const int BUZZER_FREQUENCY = 261; // Defining frequency of buzzer en Hz 2000

char customKey;

const char* colors[] = {"Rojo", "Verde", "Azul"};

void setup() {

lcd.init(); // Initializing LCD Display

//lcd.begin();

keypad.begin();

lcd.clear(); // clearing LCD display

lcd.backlight(); // turns the LCD display backlight on

pinMode(BUZZER_PIN, OUTPUT);

pinMode(LED_RED_PIN, OUTPUT);

pinMode(LED_GREEN_PIN, OUTPUT);

pinMode(LED_BLUE_PIN, OUTPUT);

//digitalWrite (BUZZER_PIN, HIGH);

lcd.init();

lcd.backlight();

lcd.clear();

lcd.print("SELECT OF COLOR:");

delay (100);

}

void loop() {

lcd.init();

lcd.backlight();

lcd.clear();

lcd.print("1.RED");

lcd.setCursor(0, 1);

lcd.print("2.GREEN 3.BLUE");

while (true) {

customKey = keypad.getKey();

if (customKey != NO_KEY) {

tone(BUZZER_PIN, BUZZER_FREQUENCY); // Feedback del buzzer

delay(100);

noTone(BUZZER_PIN);

switch (customKey) {

case '1':

setColor(255, 0, 0); // RED

break;

case '2':

setColor(0, 255, 0); // GREEN

break;

case '3':

setColor(0, 0, 255); // BLUE

break;

case '#':

return; // Enter para salir

case '*':

lcd.clear();

break;

default:

// Error de selección

tone(BUZZER_PIN, BUZZER_FREQUENCY * 2); // Buzz error

delay(200);

noTone(BUZZER_PIN);

break;

}

}

}

}

void setColor(int red, int green, int blue) {

analogWrite(LED_RED_PIN, red);

analogWrite(LED_GREEN_PIN, green);

analogWrite(LED_BLUE_PIN, blue);

}
