# Supplementary figures and images for: A novel viral RNA detection method based on the combined use of trans-acting ribozymes and HCR-FRET analyses
Source: PLoS One. 2024 Sep 26;19(9):e0310171. doi: 10.1371/journal.pone.0310171 (PMC11426510; doi:10.1371/journal.pone.0310171)

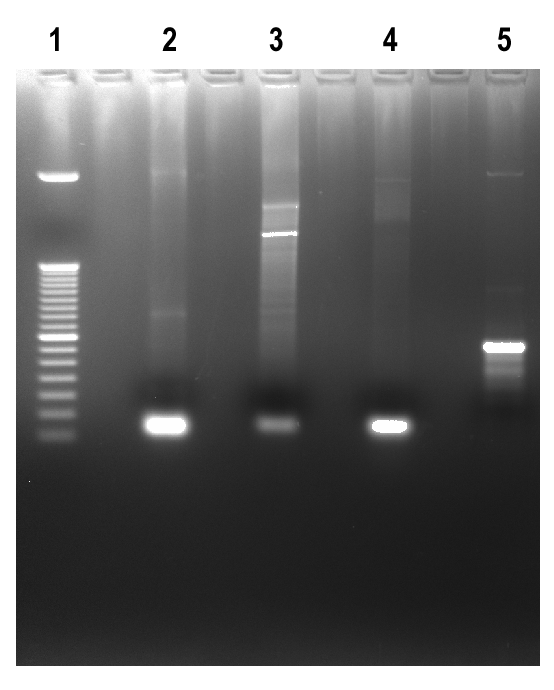

Supplement: S2 Fig — The ribozyme 1 (lane 2), ribozyme 2 (lane 3) and ribozyme 3 (lane 4) are approximately 60 nt. The target RNA is approximately 400 nt (lane 5). Invitrogen 50 bp DNA Ladder molecular marker (lane 1). (TIF) [file pone.0310171.s003.tif]

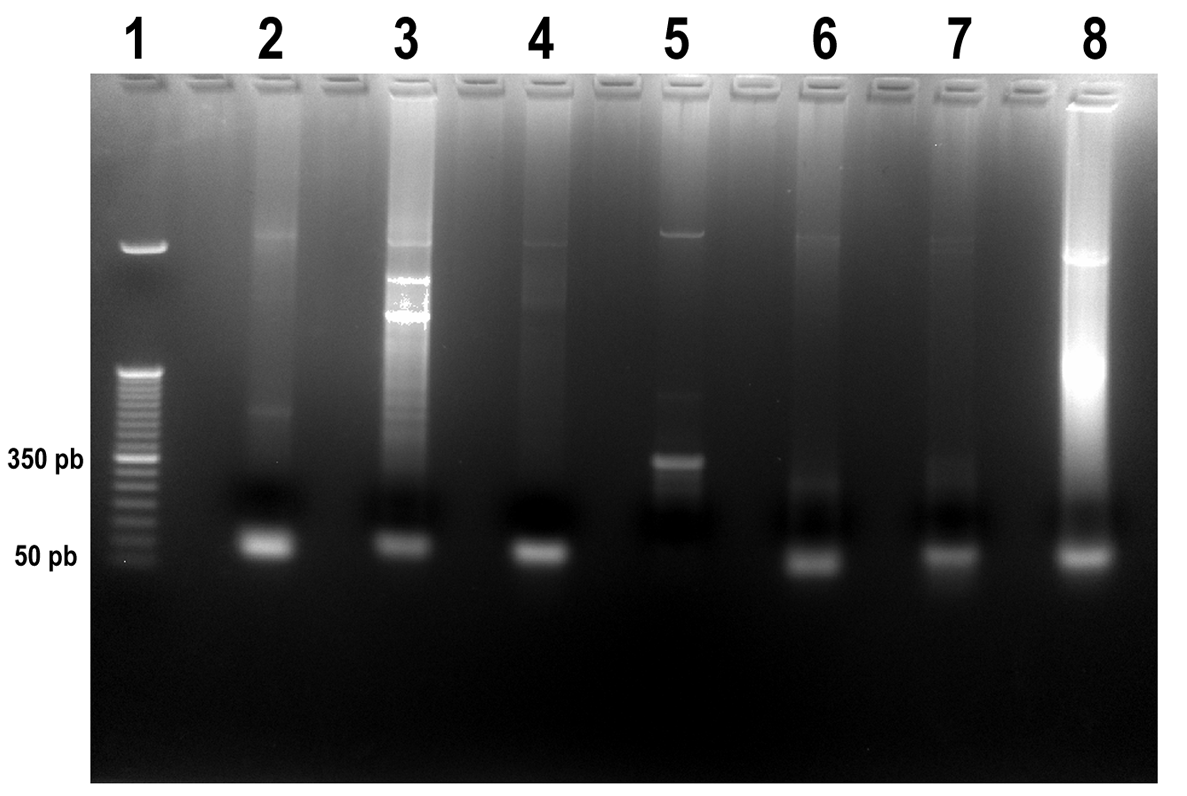

Supplement: S3 Fig — Agarose gel electrophoresis displaying RNA fragments obtained after cleavage of a 400 nt SARS-CoV-2 viral RNA target by ribozymes 1, 2 and 3. Controls for free ribozymes 1, 2, 3, and the RNA target are shown in lanes 2, 3, 4 and 5, respectively. The cleavage products are shown for ribozyme 1 (lane 6), ribozyme 3 (lane 7), and ribozyme 2 (lane 8). The target RNA is approximately 400 nt, and the ribozymes are approximately 60 bp each. Cleavage products for ribozyme 1 are 155 nt and 245 nt (lane 6), while for ribozyme 3 they are 121 nt and 279 nt (lane 7). Cleavage with ribozyme 2 resulted in several unexpected RNA fragments (lane 8). (TIF) [file pone.0310171.s004.tif]

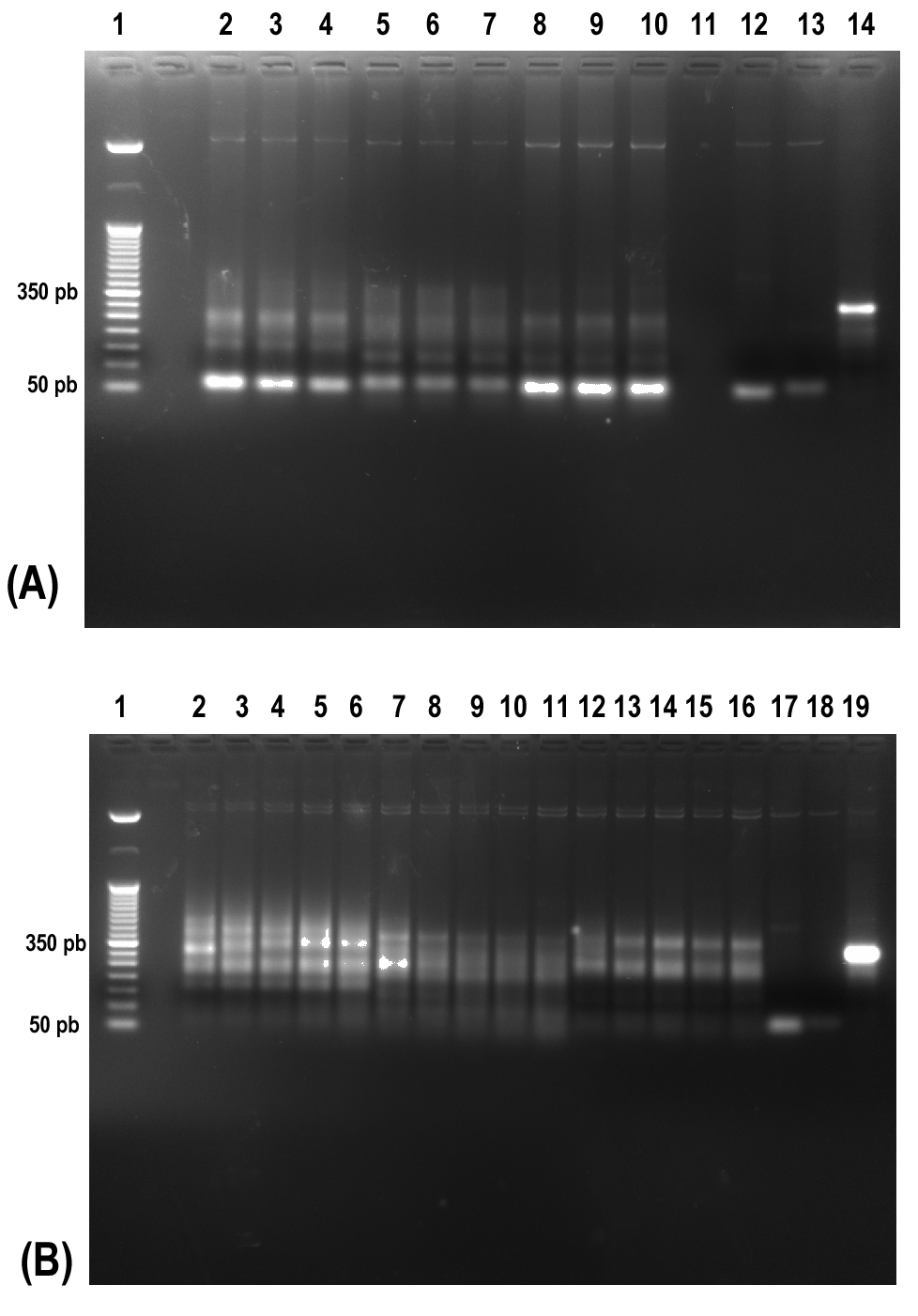

Supplement: S4 Fig — (A) Agarose gel electrophoresis displaying RNA fragments obtained after cleavage of a 400 nt SARS-CoV-2 viral RNA target by ribozyme 1 and/or ribozyme 3. The cleavage products are shown for ribozyme 1 (lanes 2–4), ribozyme 3 (lanes 5–7), and both ribozymes combined (lanes 8–10). (B) Evaluation of cleavage activity at various time points (10, 30, 60, 90, and 150 minutes) with ribozyme 1 (lanes 2–6), ribozyme 3 (lanes 7–11), and both ribozymes combined (lanes 12–16). Controls for ribozyme 1, ribozyme 3, and the RNA target are shown in lanes 12, 13, and 14 (Panel A), and lanes 17, 18, and 19 (Panel B), respectively. The target RNA is approximately 400 nt, and the ribozymes are approximately 60 bp each. Cleavage products for ribozyme 1 are 155 nt and 245 nt, while for ribozyme 3 they are 121 nt and 279 nt. The molecular marker (MM) used is the 50 bp DNA Ladder (Invitrogen), shown in lane 1 in both panels. (TIF) [file pone.0310171.s005.tif]

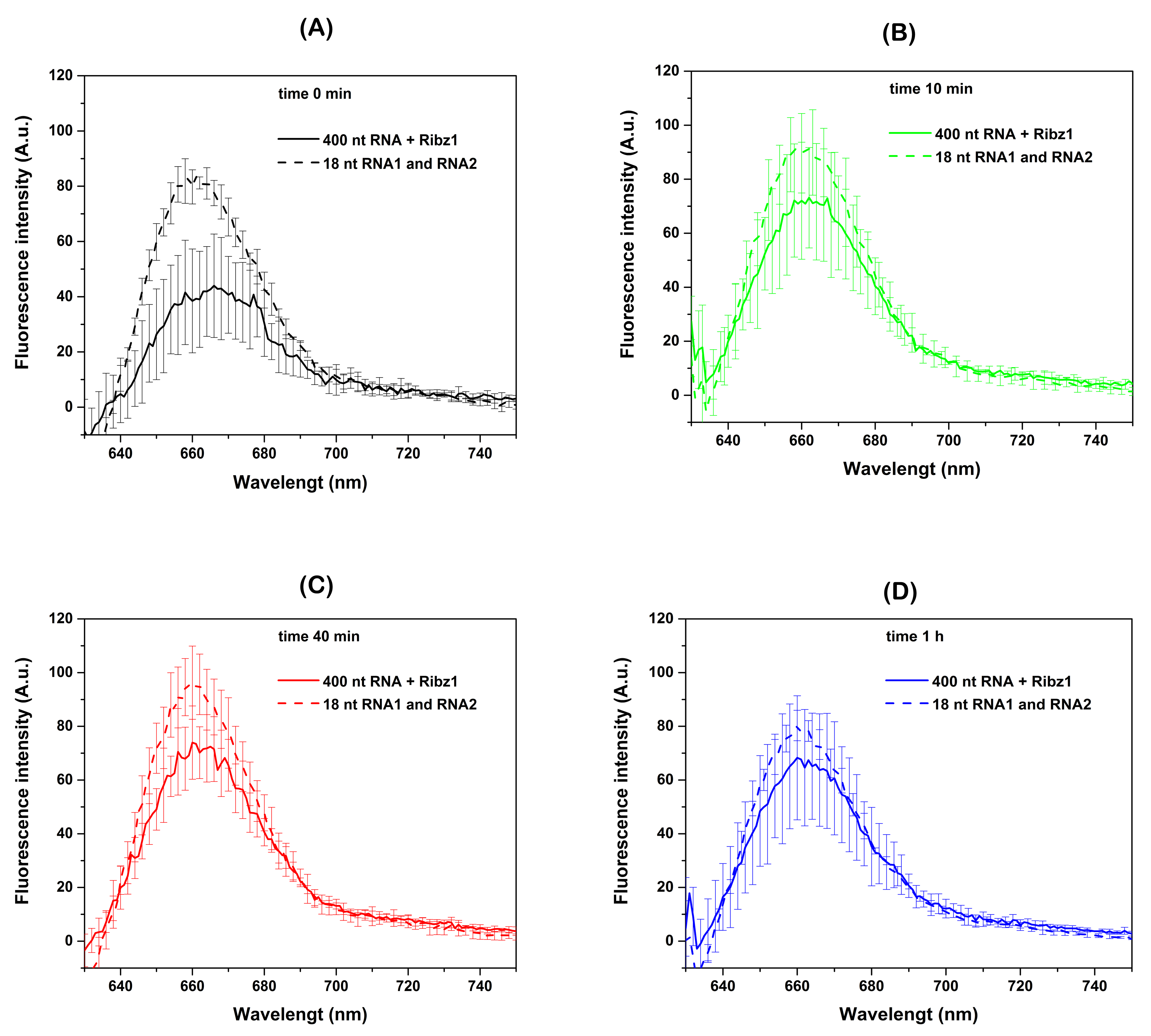

Supplement: S5 Fig — Average fluorescence intensity emission spectra from HCR-FRET between 600 nM of each DNA hairpin (H1 and H2) in 5X SSC buffer at different detection times: immediately (0 minutes), 10 minutes, 40 minutes, and one hour. Cleavage of intact 400 nt SARS-CoV-2 RNA viral fragment by ribozyme 1 was performed during 10 min. The positive control included both 18 nt initiator RNA1 and RNA2 targets at 600 nM and 600 nM of both DNA hairpins (H1 and H2). All assays were performed in technical triplicates and at least two independent experiments. Spectra were obtained by subtracting the negative control spectrum (without initiator RNA molecules). (TIF) [file pone.0310171.s006.tif]

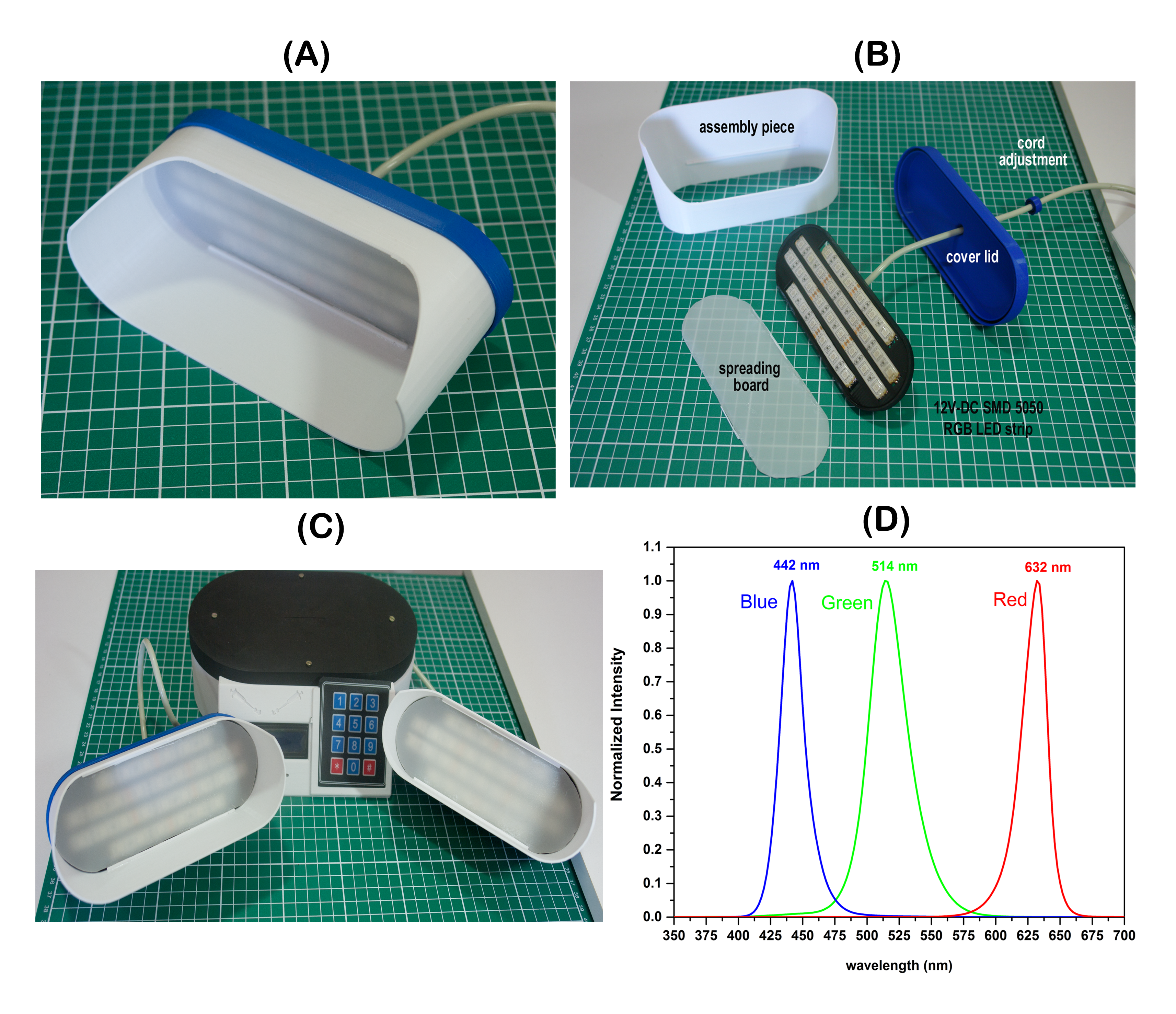

Supplement: S6 Fig — The RGB LED modules were developed and manufactured as a compact module (A) with diffuser plate (spreading board) to scatter light effectively (B). The LED modules are electronically controlled by control module through an Arduino nano V3 Atmega328 Ch340 microcontroller board, employing three TIP122 MOSFET transistors assisted by 220 Ω resistors (C). Wavelength calibration on the spectrofluorometer showed three light output spanning specific wavelengths and intensities, comprehending red (596–650 nm), green (490–560 nm), and blue (425–464 nm), with maximum wavelength of 632 nm, 514 nm, and 442 nm, respectively (D). The plastic structural components were designed using CAD software Dassault Systems SolidWorks and manufactured using 3D printing. (TIF) [file pone.0310171.s007.tif]

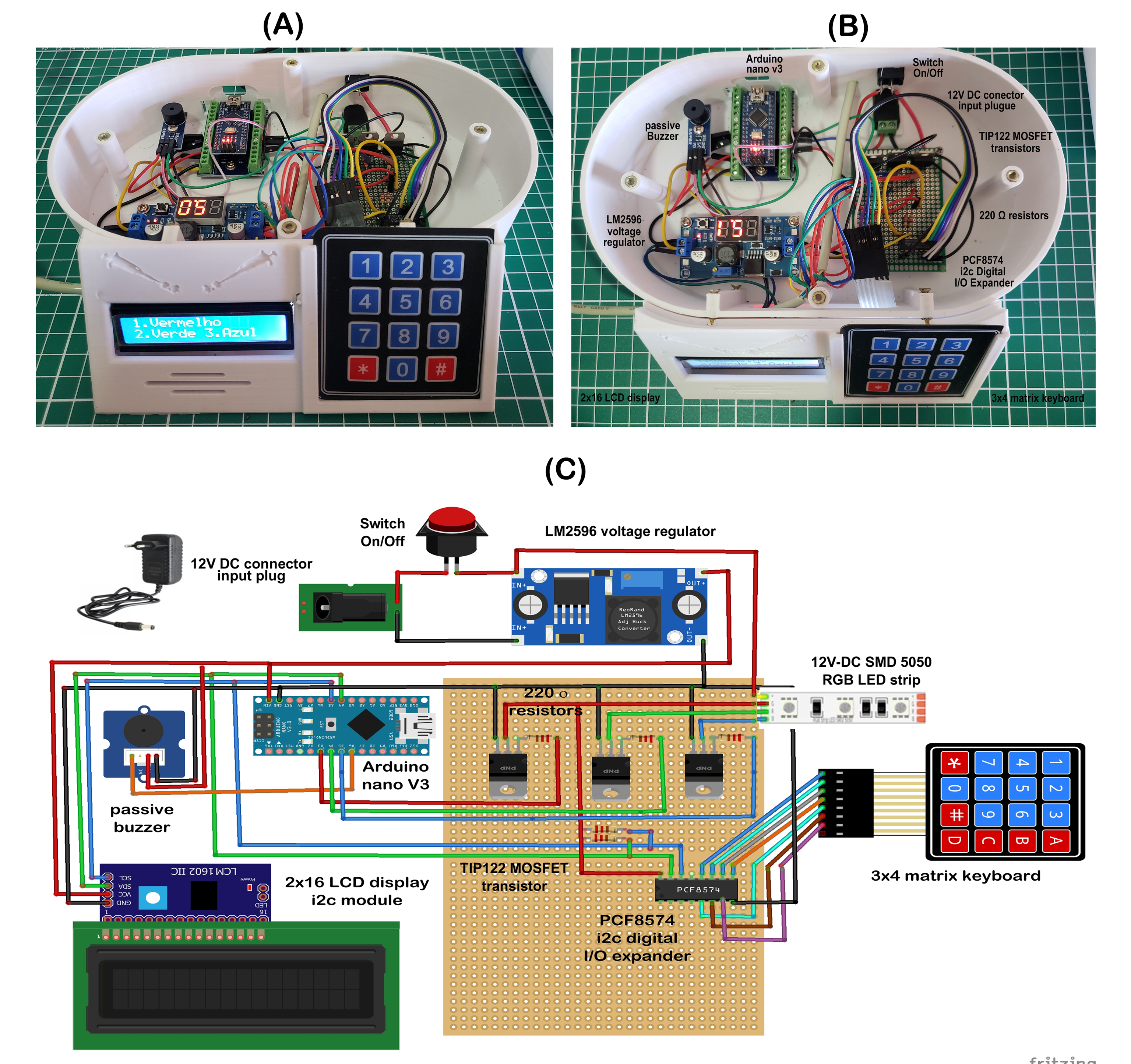

Supplement: S7 Fig — The electronic control module include an user interface module with a 2x16 LCD display with i2c module and 3x4 matrix keyboard and passive buzzer (A), a 12V DC connector plug, On/Off switch, three TIP122 MOSFET transistors assisted by 220 Ω resistors, an Arduino nano V3 Atmega328 Ch340 microcontroller board, LM2596 voltage regulator, a PCF8574 Digital I/O Expander via I2C-bus by serial clock (SCL), serial data (SDA) (B), and the control software. Electronic diagram of the control circuit using the Arduino Nano microcontroller is shown in (C). The plastic structural components were designed using CAD software Dassault Systems SolidWorks and manufactured using 3D printing. (TIF) [file pone.0310171.s008.tif]

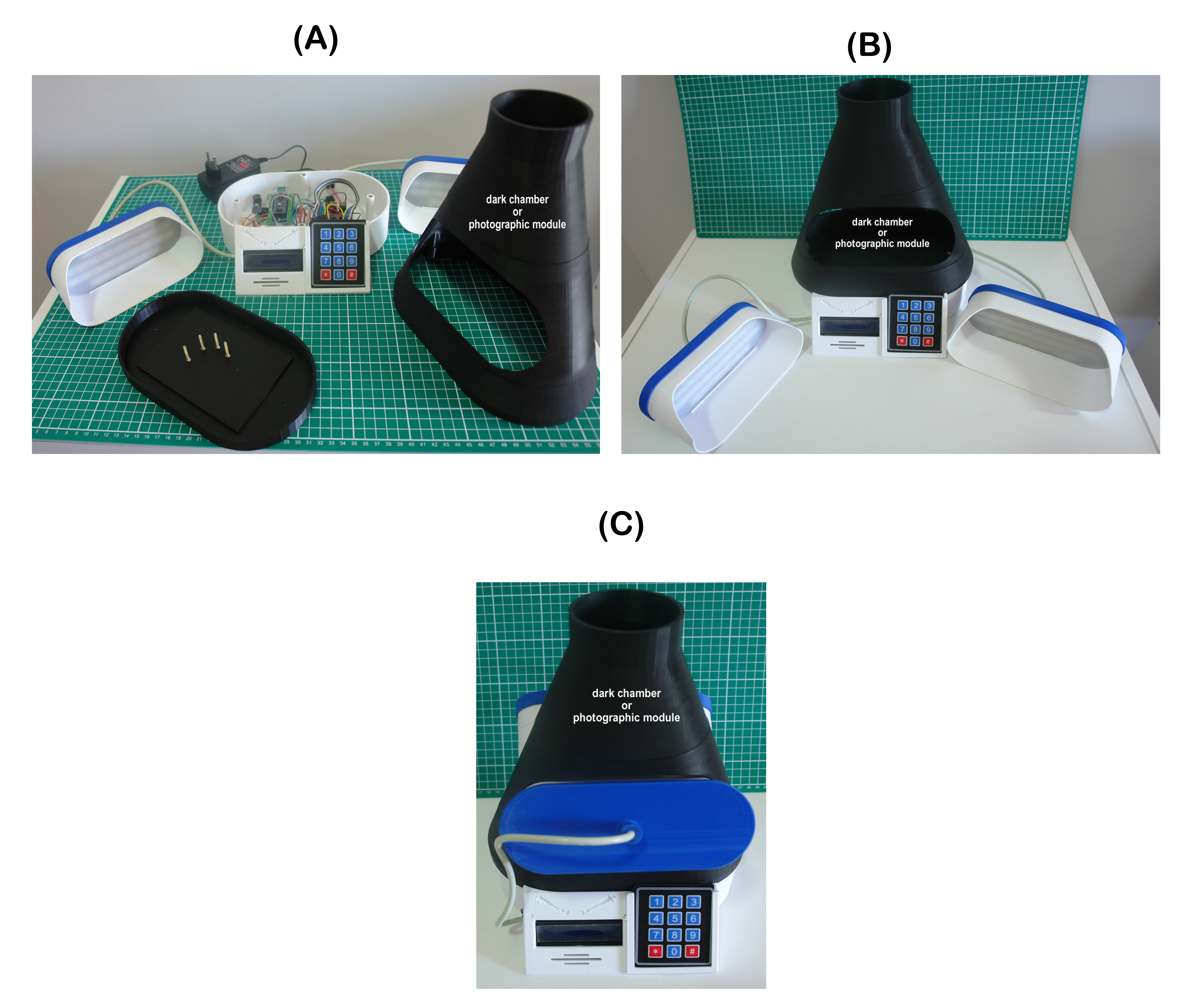

Supplement: S8 Fig — The dark chamber or photographic module designed specifically for collecting digital images (A), and its assembly on the control module (B). Complete assembly of all components or modules of the prototype DIY photo-fluorometer based on image analysis, showing the fully operational device (C). (TIF) [file pone.0310171.s009.tif]

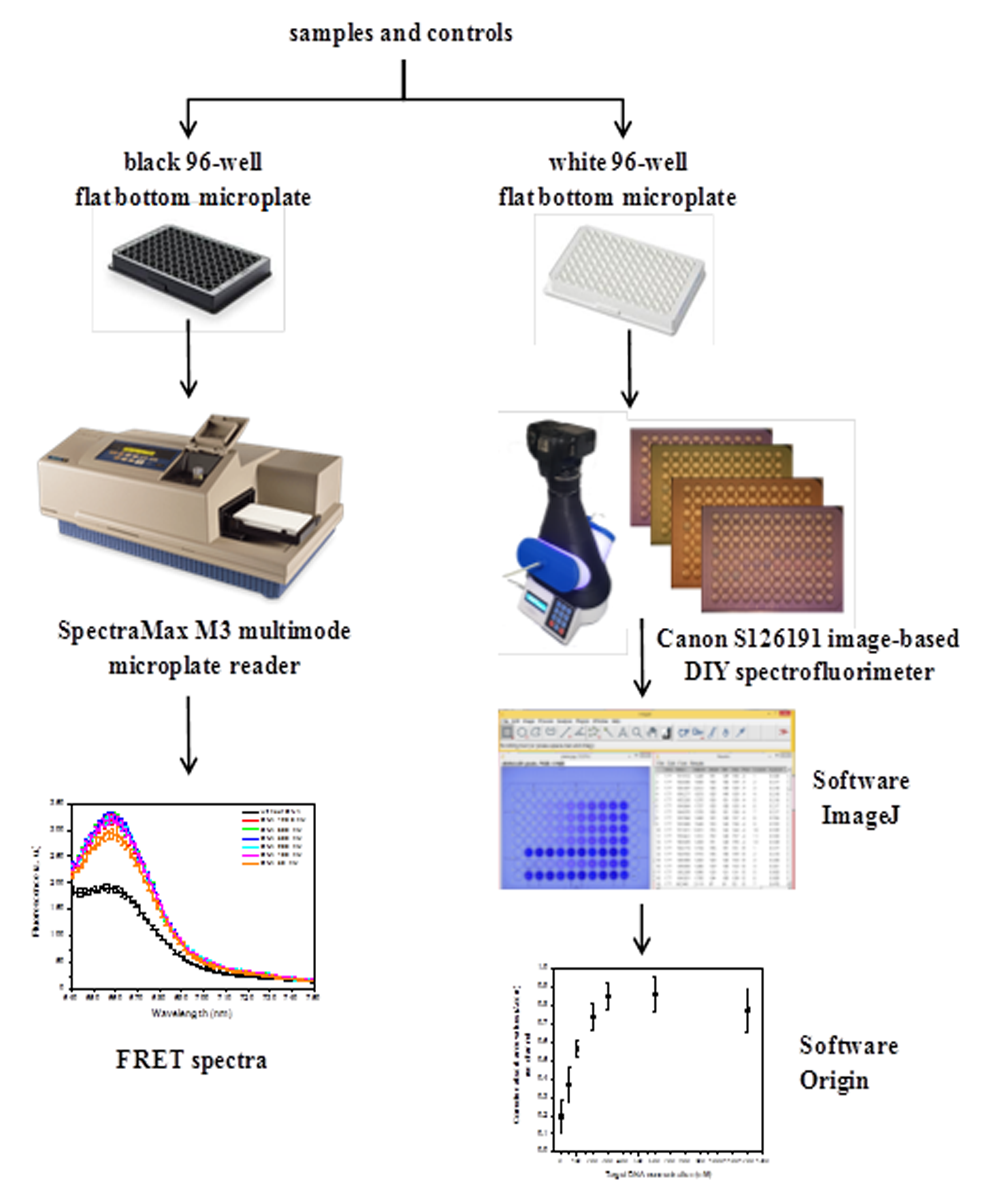

Supplement: S9 Fig — FRET reactions were prepared on black flat bottom microplate and FRET verification in each reaction was carried out on the SpectraMax M3 multimode microplate reader. These samples were transferred to fully white microplate and positioned inside the dark chamber of the device for image capture using a Canon DS126191 camera. Digital photos were acquired using each of the three RGB LEDs detection process involved digital imaging. (TIF) [file pone.0310171.s010.tif]
