# Supplementary material for: A novel viral RNA detection method based on the combined use of trans-acting ribozymes and HCR-FRET analyses
Source: PLoS One. 2024 Sep 26;19(9):e0310171. doi: 10.1371/journal.pone.0310171 (PMC11426510; doi:10.1371/journal.pone.0310171)
Supplement: Attachment — Submitted filename: Comment.docx [file pone.0310171.s011.docx]

The manuscript presents an innovative approach to viral RNA detection, focusing on the development of a method that combines trans-acting ribozymes with Hybridization Chain Reaction-Fluorescence Resonance Energy Transfer (HCR-FRET) analysis for the detection of SARS-CoV-2 viral RNA. The proposed method offers the advantages of speed, cost-effectiveness, ease of use, and does not require specialized equipment, making it particularly suitable for settings with limited resources. The research team has also designed and constructed a prototype for a Do-It-Yourself (DIY) photo-fluorometer, further exploring the potential for a simple and cost-effective point-of-care detection method based on digital image analysis. Overall, the study provides a novel molecular mechanism for the detection of viral RNA and shows promise for broad application I recommend publication of this article after addressing the following concerns.

1. Figure 1 mentioned the “Hammerhead Ribozyme”, but there is no introduction to the “Hammerhead”.

2. A comparison with existing diagnostic methods such as RT-qPCR is suggested to highlight the advantages of the proposed method in terms of sensitivity, specificity, and cost-effectiveness.

3. Some figures require improvement in clarity and labeling to ensure accurate conveyance of information.

4. SARS-CoV-2 detection in clinical throat swab samples should be investigated.

5. I recommend authors to go through another round of editing prior to submission of the revision.
