# Supplementary material for: A novel viral RNA detection method based on the combined use of trans-acting ribozymes and HCR-FRET analyses
Source: PLoS One. 2024 Sep 26;19(9):e0310171. doi: 10.1371/journal.pone.0310171 (PMC11426510; doi:10.1371/journal.pone.0310171)
Supplement: Attachment — Submitted filename: Response to Reviewers.docx [file pone.0310171.s012.docx]

July 21, 2024

**Professor Dr. Andrew Max Abaasa**

Editorial Board Member, PLOS ONE

**Re: Manuscript Ref. No.: PONE-D-24-22185**

**Title: *A novel viral RNA detection method based on the combined use of trans-acting ribozymes and HCR-FRET analyses.***

Dear Professor Abaasa,

Thank you for your prompt response and for the thorough evaluation of our manuscript. We are grateful to you and the reviewers for your careful reading and constructive comments.

We appreciate the detailed feedback provided by the reviewers, which has undoubtedly enhanced the quality of our manuscript. We have carefully considered and addressed all comments and suggestions from the two reviewers. As a result, we have revised and modified the manuscript accordingly.

We believe that the revisions made have significantly improved the clarity and overall quality of our work, and we hope that it now meets the standards for publication in PLOS ONE.

We would like to update our financial disclosure due to the close proximity to the deadline of our Project 7174/FUB/EMENDA/DPI/COPEI-UnB, it is possible that the payment charges will be totally or partially financed by a new grant applied to the *Decanato de Pesquisa e Inovação* (DPI/PPG/BCE-UnB) and the Molecular Biology Graduate Program (PPG-BioMol-UnB) from the University of Brasilia.

Enclosed, you will find our point-by-point responses to the reviewers' comments, highlighted in blue, along with the revised manuscript and figures files submitted through the Editorial Manager PLOS ONE system.

Thank you once again for your time and effort in reviewing our manuscript. We look forward to your favorable consideration for publication.

With best regards,

Dra.Cíntia Marques Coelho

**Responses to the editors comments:**

***1. Please note that funding information should not appear in any section or other areas of your manuscript. We will only publish funding information present in the Funding Statement section of the online submission form. Please remove any funding-related text from the manuscript.***

We agree with the editors and have removed the following funding information from the Acknowledgments section in manuscript (lines 765-778).

*We thank support agencies: International support to beneficiary C.M.C. from Just One Giant Lab (JOGL), France. National support to F.T. from Conselho Nacional de Desenvolvimento Científico e Tecnológico (CNPq, process 406375/2022-4), Brazil. National support from Fundação de Apoio à Pesquisa do Distrito Federal (FAPDF, Project No 00193-00000781/2021-19), Brasília, DF, Brazil to S.M.F. that include a technical scholarship to L.C.S., and post-doctoral scholarships to A.V.G.; I.C.M.O.; and A.S.B. National support as Graduation study scholarship to P.F.Q. from Decanato de Pesquisa e Inovação e Decanato de Pós-Graduação (DPI/DPG) Molecular Biology program, from University of Brasília, and Master post graduation scholarship to L.F.S. from DPI/DPG and Coordenação de Aperfeiçoamento de Pessoal de Nível Superior (CAPES). National support to C.M.C. from Comitê de Pesquisa, Inovação e Extensão de combate à COVID-19, Decanato de Pesquisa e Inovação Decanato de Extensão from University of Brasília (Project 7174/FUB/EMENDA/DPI/COPEI-UnB), Brazil. The funders had no role in study design, data collection and analysis, decision to publish, or preparation of the manuscript.*

We replaced the text by:

*We thank following support agencies: Just One Giant Lab (JOGL), France; Conselho Nacional de Desenvolvimento Científico e Tecnológico (CNPq), Brazil; Fundação de Apoio à Pesquisa do Distrito Federal (FAPDF), Brazil; Decanato de Pesquisa e Inovação e Decanato de Pós-Graduação (DPI/DPG) Molecular Biology program, from University of Brasília; Coordenação de Aperfeiçoamento de Pessoal de Nível Superior (CAPES); Comitê de Pesquisa, Inovação e Extensão de combate à COVID-19, and Decanato de Pesquisa e Inovação Decanato de Extensão from University of Brasília, Brazil. We thank Roberto Togawa for his assistance with the computational alignment analysis.*

***2. We note that you have included the phrase “data not shown” in your manuscript. Unfortunately, this does not meet our data sharing requirements. PLOS does not permit references to inaccessible data. We require that authors provide all relevant data within the paper, Supporting Information files, or in an acceptable, public repository. Please add a citation to support this phrase or upload the data that corresponds with these findings to a stable repository (such as Figshare or Dryad) and provide and URLs, DOIs, or accession numbers that may be used to access these data. Or, if the data are not a core part of the research being presented in your study, we ask that you remove the phrase that refers to these data.***

We agree with the editors. Two phrases were presented in the manuscript and have been deleted.

The first instance, in lines 466-468, was updated to include a supplementary figure presenting the results.

*Considering that ribozyme 2 was transcribed at a lower concentration and its cleavage activity assays resulted in several unexpected RNA fragments (data not shown), this ribozyme was not used in subsequent assays.*

by

*Considering that ribozyme 2 was transcribed at a lower concentration (S2 Fig) and its cleavage activity assays resulted in several unexpected RNA fragments (S3 Fig), this ribozyme was not used in subsequent assays.*

S3 Figure Legend was included:

***S3 Figure. Agarose Gel Electrophoresis of Ribozymes Catalytic Assay.*** *Agarose gel electrophoresis displaying RNA fragments obtained after cleavage of a 400 nt SARS-CoV-2 viral RNA target by ribozymes 1, 2 and 3. Controls for free ribozymes 1, 2, 3, and the RNA target are shown in lanes 2, 3, 4 and 5, respectively. The cleavage products are shown for ribozyme 1 (lane 6), ribozyme 3 (lane 7), and ribozyme 2 (lane 8). The target RNA is approximately 400 nt, and the ribozymes are approximately 60 bp each. Cleavage products for ribozyme 1 are 155 nt and 245 nt (lane 6), while for ribozyme 3 they are 121 nt and 279 nt (lane 7). Cleavage with ribozyme 2 resulted in several unexpected RNA fragments (lane 8).*

The second instance, lines 662-665 were updated to include an additional panel in Figure 11, presenting the results, and the main text was revised accordingly:

*When excited with red light (596-650 nm), a highly saturated intensity is observed in the red channel of the digital photo of the wells, possibly due to scattering of the red excitation light (data not shown).* *The complete saturation of this channel's intensity difficult to process the images in the wells.*

by

*When excited with red light (596-650 nm), a highly saturated intensity is observed in the red channel of the digital photo of the wells, possibly due to scattering of the red excitation light. The complete saturation of this channel's intensity made it difficult to process the images in the wells, resulting in intensities values corrected by the software very close to zero (Fig 11B).*

Figure 11D Legend was included Lines 655-656:

*The results of excitation with blue light are shown in panels (B) and with green light (C).*

by

*The results of excitation with red, blue, and green light are shown in panels (B), (C), (D), respectively.*

***3. PLOS ONE now requires that authors provide the original uncropped and unadjusted images underlying all blot or gel results reported in a submission’s figures or Supporting Information files. This policy and the journal’s other requirements for blot/gel reporting and figure preparation are described in detail at https://journals.plos.org/plosone/s/figures#loc-blot-and-gel-reporting-requirements and https://journals.plos.org/plosone/s/figures#loc-preparing-figures-from-image-files. When you submit your revised manuscript, please ensure that your figures adhere fully to these guidelines and provide the original underlying images for all blot or gel data reported in your submission. See the following link for instructions on providing the original image data:*** [***https://journals.plos.org/plosone/s/figures#loc-original-images-for-blots-and-gels***](https://journals.plos.org/plosone/s/figures#loc-original-images-for-blots-and-gels)***.***

We agree with the editors and have modified Figure 5 according to the guidelines. Labels within the image were removed, and consequently, information regarding the order of sample application was transferred to the figure legend. The legend for Figure 5 was thus modified as follows:

Line (470-475):

***Figure 5. Agarose gel electrophoresis of Ribozyme catalytic assay products.*** *Ribozyme catalytic assay showing the RNA fragments obtained after ribozyme 1 and 3 cleavage of the 400 nt target RNA molecule analyzed in 2% agarose gel electrophoresis. The target RNA is approximately 400 nt and the ribozymes of approximately 60 nt. RNA cleaved products for ribozyme 1 was 155 nt and 245 nt and for ribozyme 3 was 121 nt and 279 nt. Molecular Marker (MM) was the 50 bp DNA Ladder (Invitrogen).*

by

***Figure 5. Agarose gel electrophoresis of Ribozyme catalytic assay products.*** *Ribozyme catalytic assay showing the RNA fragments obtained after ribozyme 1 (lanes 2-5) and ribozyme 3 (lanes 6-9) cleavage of the 400 nt target RNA molecule analyzed in 2% agarose gel electrophoresis. The target RNA is approximately 400 nt (lane 10), and the ribozyme 1 (lane 11) and ribozyme 3 (lane 12) of approximately 60 nt. RNA cleaved products for ribozyme 1 was 155 nt and 245 nt and for ribozyme 3 was 121 nt and 279 nt. Molecular Marker (MM) (lane 1) was the 50 bp DNA Ladder (Invitrogen).*

We modified S2 Figure according to the guidelines. Labels within the image were removed, and consequently, information regarding the order of sample application was transferred to the figure legend. The legend for Figure S2 was thus modified as follows:

Line (974-976):

***S2 Figure. Target RNA and ribozymes transcribed in vitro in 2% agarose gel electrophoresis.*** *The target RNA is approximately 400 nt and the ribozymes are approximately 60 nt. Invitrogen 50 bp DNA Ladder molecular marker.*

by

***S2 Figure. Target RNA and ribozymes transcribed in vitro in 2% agarose gel electrophoresis.*** *The ribozyme 1 (lane 2), ribozyme 2 (lane 3) and ribozyme 3 (lane 4) are approximately 60 nt. The target RNA is approximately 400 nt (lane 5). Invitrogen 50 bp DNA Ladder molecular marker (lane 1).*

We modified S3 Figure as S4 Figure according to the guidelines. Labels within the image were removed, and consequently, information regarding the order of sample application was transferred to the figure legend. The legend for Figure S4 was thus modified as follows:

Line (977-983):

***S3 Figure. Agarose gel electrophoresis of dried ribozymes catalytic assay.*** *Agarose gel electrophoresis showing the RNA fragments obtained after ribozyme 1 and/or ribozyme 3 cleavage of the 400 nt SARS-CoV-2 viral RNA target. The size of the target RNA is approximately 400 nt, and the ribozymes are approximately 60 bp. Cleavage products for ribozyme 1 were 155 nt and 245 nt, while for ribozyme 3 they were 121 nt and 279 nt. The molecular marker (MM) used was the 50 bp DNA Ladder (Invitrogen). Catalytic assay before and after dried for each one of the ribozymes (A). Evaluation of the cleavage activity at different times: 10, 30 60, 90 and 150 minutes (B).*

by

***Figure S4. Agarose Gel Electrophoresis of Dried Ribozymes Catalytic Assay.*** *(A) Agarose gel electrophoresis displaying RNA fragments obtained after cleavage of a 400 nt SARS-CoV-2 viral RNA target by ribozyme 1 and/or ribozyme 3. The cleavage products are shown for ribozyme 1 (lanes 2-4), ribozyme 3 (lanes 5-7), and both ribozymes combined (lanes 8-10). (B) Evaluation of cleavage activity at various time points (10, 30, 60, 90, and 150 minutes) with ribozyme 1 (lanes 2-6), ribozyme 3 (lanes 7-11), and both ribozymes combined (lanes 12-16). Controls for ribozyme 1, ribozyme 3, and the RNA target are shown in lanes 12, 13, and 14 (Panel A), and lanes 17, 18, and 19 (Panel B), respectively. The target RNA is approximately 400 nt, and the ribozymes are approximately 60 bp each. Cleavage products for ribozyme 1 are 155 nt and 245 nt, while for ribozyme 3 they are 121 nt and 279 nt. The molecular marker (MM) used is the 50 bp DNA Ladder (Invitrogen), shown in lane 1 in both panels.*

The remaining supplementary figures in the manuscript, along with their legends, were renumbered.

**Responses to the reviewer 1 comments:**

In this manuscript, the authors developed an assay for detecting SARS CoV-2 RNA viral based on trans-acting ribozymes and FRET-based hybridization of fluorescent DNA hairpins. The target RNA is specifically recognized and cleaved by ribozymes, releasing an initiator fragment that triggers a hybridization chain reaction (HCR) with DNA hairpins containing fluorophores, leading to a FRET process. The manuscript has abundant experimental data, but remains some questions to be addressed.

1. Please simplify the abstract section. It is suggested not to include too much research background in the abstract.

2. The introduction section does not adequately demonstrate the advancements of the HCR-based assay. The authors should discuss the progress made in this area and highlight the key challenges.

3. The schematic figures are simple and need to be improved to meet the journal's standards.

4. The details of gel electrophoresis procedures should be provided.

5. This method utilizes the trans-acting ribozymes to recognize SARS-CoV-2 RNA. Is it promising to identify single nucleotide mutations in viral RNA?

6. The authors indicated that this method is cost-effective and user-friendly. Therefore, the detection cost and preprocessing requirements of this method need to be discussed.

***1. Please simplify the abstract section. It is suggested not to include too much research background in the abstract.***

We agree with the reviewer and have simplified the abstract section by removing the following text about background knowledge (lines 28-32):

*These include the need for refrigeration, specialized equipment, and professional expertise. Therefore, there is a need for a diagnostic method that is rapid, cost-effective, user-friendly, and does not require specialized equipment compared to other techniques. Such a method would be extremely useful in resource-limited settings, enhancing readiness for future epidemics and pandemics.*

***2. The introduction section does not adequately demonstrate the advancements of the HCR-based assay. The authors should discuss the progress made in this area and highlight the key challenges.***

We agree with the reviewer and have replaced the following text (Lines 78-88) in the Introduction section:

*This methodology involves two hairpin DNA probes, each labeled with fluorophores, containing complementary regions that, upon encountering a target initiator, concatenate by hybridization to form an elongated double-stranded DNA structure. This enables the detection of the acid nucleic target via situ hybridization and through the enhanced amplification fluorescence signals (14-16). However, this HCR method requires a washing step to removal non-binding probes. To optimize this stage and broaden the methodology's applicability, a Fluorescence Resonance Energy Transfer (FRET) reaction has been incorporated into the HCR approach (17-19). The FRET operates based on the transference of energy fluorescence between donor-acceptor molecular pairs, making it a highly efficient method for diagnostic applications. It offers the advantage of single-step execution and specific identification of the target molecules using a fluorophore-labeled molecular probe and smartphones (20).*

by:

*The concept of HCR was introduced by Dirks and Pierce in 2004 (15). In HCR, stable DNA monomers are assembled only in the presence of a target DNA or RNA fragment. This process involves two stable DNA hairpins, containing complementary regions, which coexist in solution until the initiator strands trigger a hybridization cascade, forming nicked double helices. The molecular weight of HCR products varies inversely with initiator concentration, allowing DNA to act as an amplifying transducer for biosensing applications (15, 16). Despite its advantages, HCR faces challenges that need to be addressed. Optimizing reaction conditions for consistent performance, improving the speed of the HCR process, and developing robust protocols for multiplexed detection are key areas needing advancement.*

*To optimize it and broaden the methodology's applicability, a Fluorescence Resonance Energy Transfer (FRET) reaction has been incorporated into the HCR approach. The FRET operates based on the transference of energy fluorescence between donor-acceptor molecular pairs, making it a highly efficient method for diagnostic applications (16-18). This methodology HCR-FRET has been applied to identify and image mRNA in situ using fluorescence microscopy, and to detect tumor-related mRNA in single cells and tissue sections with high sensitivity (19, 20). This ability is promising for early cancer diagnosis by distinguishing between cancer and normal cells (16). Thus it, offers the advantage of single-step execution and specific identification of the target molecules using a fluorophore-labeled molecular probe and smartphones (20).*

New references have been included and updated.

***3. The schematic figures are simple and need to be improved to meet the journal's standards.***

Thank you for your valuable feedback regarding the schematic figures. In response, we have revised the figures to ensure they meet the journal's standards and comply with the guidelines for authors. The updated figures are now formatted as high-quality .TIFF files with LZW compression. They are in RGB color mode (8 bit/channel), with dimensions ranging from 789 to 2250 pixels in width (at 300 dpi) and a maximum height of 2625 pixels (at 300 dpi). The resolution of the figures are 600 dpi. All text within the figures is set in Arial font, ranging from 8 to 12 points in size. Additionally, multi-panel figures have been combined into a single file for clarity and ease of review.

***4. The details of gel electrophoresis procedures should be provided.***

We appreciate the reviewer's suggestion and have added the electrophoresis procedure details to the Materials and Methods section (at lines 169):

*Horizontal agarose gel electrophoresis was performed using RNase-free low melting agarose 2% (w/v) in a 15 x 15 cm UV-transparent tray system with PowerPac™ Basic Power Supply (Bio-Rad Laboratories, Inc., Hercules, California, USA). The electrophoresis was conducted with 1x Tris/Acetic Acid/EDTA (TAE) nucleic acid electrophoresis buffer solution at pH 8.3, at a constant 80 Volts. Subsequently, the gels were stained with 0.01 mg/mL of ethidium bromide (35).*

Reference 39 was included.

***5. This method utilizes the trans-acting ribozymes to recognize SARS-CoV-2 RNA. Is it promising to identify single nucleotide mutations in viral RNA?***

We appreciate the reviewer's question regarding the use of trans-acting ribozymes for the detection of SARS-CoV-2 RNA and their potential for identifying single nucleotide mutations in viral RNA.

To address this, our strategy for selecting target ribozyme sequences was designed to ensure specific identification of SARS-CoV-2. We focused on a 400 nt fragment previously used by other authors to develop the test for SARS-CoV-2 identification (Reference 30 in the first manuscript: *Hou T, Zeng W, Yang M, Chen W, Ren L, Ai J, et al. Development and evaluation of a rapid CRISPR-based diagnostic for COVID-19. PLoS pathogens. 2020;16(8):e1008705*). The fragment is within the ORF1ab region (YP_009724389.1, positions 11,459-11,858) corresponding to the non-structural protein 6 (Nsp6). Nsp6 is a highly conserved membrane protein with six transmembrane domains, essential for viral replication. Due to its crucial role, it exhibits high sequence conservation among SARS-CoV-2 strains.

In our study, we aligned this 400 nt fragment with 16,000 consensus sequences of SARS-CoV-2, achieving over 98% identity. This high level of conservation makes it an ideal target for ribozyme design. We selected three ribozymes targeting this region and evaluated their specificity by aligning their target sequences with genomes of related coronaviruses (SARS-CoV, MERS-CoV, and other common human coronaviruses).

The results showed significant differences in nucleotide sequences between SARS-CoV-2 and other coronaviruses, confirming the specificity of our selected ribozymes. For instance:

Ribozyme 1 (22 nts):

- Genome-229: 10 nucleotide differences
- Bat SARS-like genome: 5 nucleotide differences
- HKU1 genome: 12 nucleotide differences
- MERS control and MERS NC genomes: 9 nucleotide differences
- NL63 genome: 10 nucleotide differences
- OC43 genome: 10 nucleotide differences
- SARS Tor 2 genome: 8 nucleotide differences

Ribozyme 2 (30 nts):

- Genome-229: 13 nucleotide differences
- Bat SARS-like genome: 7 nucleotide differences
- HKU1 genome: 13 nucleotide differences (including 1 deletion and 1 insertion)
- MERS control and MERS NC genomes: 12 nucleotide differences
- NL63 genome: 12 nucleotide differences
- OC43 genome: 15 nucleotide differences (including 1 deletion and 1 insertion)
- SARS Tor 2 genome: 8 nucleotide differences

Ribozyme 3 (23 nts):

- Genome-229: 4 nucleotide differences
- Bat SARS-like genome: 2 nucleotide differences
- HKU1 genome: 7 nucleotide differences
- MERS control and MERS NC genomes: 6 nucleotide differences
- NL63 genome: 7 nucleotide differences
- OC43 genome: 7 nucleotide differences
- SARS Tor 2 genome: 2 nucleotide differences

These alignments suggested that our ribozymes are highly specific to SARS-CoV-2 and differ significantly from other related viruses. This specificity indicates that our ribozyme-based method could be promising for identifying single nucleotide mutations in SARS-CoV-2 RNA, enhancing its potential utility in detecting and monitoring viral mutations. However, experimental assays are necessary to confirm this hypothesis.

Additionally, to better clarify the analysis carried out, we replaced the following text of the manuscript (lines 313-318):

*To confirm that the target sequence represents a consensus to the SARS-CoV-2 variants, genomic alignment analysis of different viral strains was performed and available (https://figshare.com/account/articles/25922725). The selected conserved sequence was, then, compared to sequences from other human viruses such as the SARS and MERS families, to withdraw the sequences that are also consensual for these other viruses.*

*by*

*The target sequence is a 400 nt fragment within the ORF1ab region (YP_009724389.1, positions 11,459-11,858) corresponding to the non-structural protein 6 (Nsp6) (https://www.ncbi.nlm.nih.gov/gene/43740578). Nsp6 is a highly conserved membrane protein with six transmembrane domains, essential for viral replication (36, 37). The alignment with 16,000 consensus sequences of SARS-CoV-2 achieved over 98% identity (https://figshare.com/account/articles/25922725), making the 400 nt fragment an ideal target for ribozyme design. Three ribozymes targeting this region were selected and their specificity were evaluated by aligning target sequences with the genomes of related coronaviruses (SARS-CoV, MERS-CoV, and other common human coronaviruses). The results revealed significant nucleotide sequence differences between SARS-CoV-2 and the other coronaviruses, indicating the specificity of selected ribozymes.*

***6. The authors indicated that this method is cost-effective and user-friendly. Therefore, the detection cost and preprocessing requirements of this method need to be discussed.***

We appreciate the reviewer's observation. However, the results presented in the manuscript do not aim to evaluate whether the method is cost-effective and user-friendly. These considerations were due to the costs of establishing the methodology and creating prototypes being higher than the cost of manufacturing a commercial kit, mainly in the proof-of-concept and prototyping phase. We estimate that a molecular reaction using HCR-FRET costs about $8-10 under prototype experimental conditions. However, in a manufactured kit, costs would be significantly lower. Therefore, in this manuscript, we emphasize the importance of developing cost-effective and user-friendly methods for long-term global emerging infectious diseases.

We believe that the method described here has potential for the mentioned characteristics based on certain points below. Using a DIY photo-fluorometer prototype allowed us to explore the development of a simple and cost-effective point-of-care detection method, based on digital image analysis. This assertion is supported by comparing the cost of prototyping the device, which can be used without extensive specialized training, with the cost of a spectrofluorometer, or plate-reading spectrofluorometer, and with the specialized knowledge required to operate it. The components and manufacturing cost of the prototype are in the range of a few tens of dollars, compared to the tens of thousands of dollars for a spectrofluorometer, not including the specialized training for the personnel who operate it.

In this context, we revised the manuscript to avoid drawing conclusions about the cost-effectiveness and user-friendliness of the methodology. As previously mentioned in our response to the reviewer's first observation, we have removed the following text from the abstract section (lines 28-32):

*These include the need for refrigeration, specialized equipment, and professional expertise. Therefore, there is a need for a diagnostic method that is rapid, cost-effective, user-friendly, and does not require specialized equipment compared to other techniques. Such a method would be extremely useful in resource-limited settings, enhancing readiness for future epidemics and pandemics.*

Additionally, in conclusion section we replaced the following text of the manuscript (lines 743-751):

*The possibilities of future pandemics, like COVID-19, highlight the critical need for simple and accessibly diagnostic tests to ensure rapid and widespread detection. Investing in user-friendly, cost-effective diagnostic methods is essential for global preparedness and timely response to emerging infectious diseases. Even after the World Health Organization (WHO) declared the end of the COVID-19 public health emergency, the emergence of the EG.5 variant of SARS-CoV-2 has led to new outbreaks, underscoring the importance of sustained vigilance (49). While RT-qPCR remains the gold standard for COVID-19 and other viral diagnoses, our proposed methodology offers a promising alternative that addresses resource limitations, refrigeration challenges, and the need for specialized expertise and equipment.*

by

*Considering future pandemics such as COVID-19, it is a necessary to develop simple, cost-effective, user-friendly, accessible, and quickly adaptable diagnostic methods for new viruses and emerging pathogens. In this context, the development of diagnostic methods based on HCR-FRET can result in high-throughput initial screening. In the case of COVID-19, even after the World Health Organization (WHO) declared the end of the COVID-19 public health emergency, the emergence of the EG.5 variant of SARS-CoV-2 led to new outbreaks, highlighting the importance of developing new technologies (56). It is important to emphasize that the proposed methodology is not intended to replace highly specific and sensitive methods based on enzymatic amplification thermocycles, such as PCR and RT-qPCR. Rather, it can serve as a foundation for alternative viral diagnostic methods that address resource limitations and the need for specialized expertise and equipment.*

and the following text of the manuscript (lines 752-754):

*In this study, a novel molecular method was conceived and developed for detecting SARS-CoV-2 RNA viral based on trans-acting ribozymes and FRET-based hybridization of fluorescent DNA hairpins.*

by

*In this study, we developed a novel molecular method for detecting viral genome fragments. As a proof of concept, we used SARS-CoV-2 RNA viral fragments. The method is based on trans-acting ribozymes and FRET-based hybridization of fluorescent DNA hairpins.*

Other minor corrections and modifications in the manuscript:

1. Line 365-366:

For the HCR-FRET assay three different final concentrations of the 18 nt initiator DNA molecule …

by

For the HCR-FRET assay, three different concentrations of the 18 nt initiator DNA molecule ….

2. Legenda Figure 3: Line 404-405:

The average fluorescence spectra emission (n = three independent experiments) were measured using the Varioskan …

by

Fluorescence emission spectra (n = three independent experiments) were recorded using the Varioskan …..

3. Legenda Figure 7: Line 507-509:

… while a single cleave by ribozyme 3 generates the segments of 121 nt and 279 nt (C). HCR-FRET may occur on 245 nt and 279 nt segments by the first DNA hairpins H1/H2 set.

by

… while a single cleavage by ribozyme 3 generates the segments of 121 nt and 279 nt (C). HCR-FRET may occur in segments of 245 nt and 279 nt by the first set of H1/H2 DNA hairpins.

4. Line 595:

… diagnostic process, relative to others methods, by ….

by

… diagnostic process, relative to other methods, by ….

5. Legenda Figure 11: Line 650:

The readings was carried out using the SpectraMax ….

by

The readings were carried out using the SpectraMax ….

**Responses to the reviewer 2 comments:**

The manuscript presents an innovative approach to viral RNA detection, focusing on the development of a method that combines trans-acting ribozymes with Hybridization Chain Reaction-Fluorescence Resonance Energy Transfer (HCR-FRET) analysis for the detection of SARS-CoV-2 viral RNA. The proposed method offers the advantages of speed, cost-effectiveness, ease of use, and does not require specialized equipment, making it particularly suitable for settings with limited resources. The research team has also designed and constructed a prototype for a Do-It-Yourself (DIY) photo-fluorometer, further exploring the potential for a simple and cost-effective point-of-care detection method based on digital image analysis. Overall, the study provides a novel molecular mechanism for the detection of viral RNA and shows promise for broad application I recommend publication of this article after addressing the following concerns.

1. Figure 1 mentioned the “Hammerhead Ribozyme”, but there is no introduction to the “Hammerhead”.

2. A comparison with existing diagnostic methods such as RT-qPCR is suggested to highlight the advantages of the proposed method in terms of sensitivity, specificity, and cost-effectiveness.

3. Some figures require improvement in clarity and labeling to ensure accurate conveyance of information.

4. SARS-CoV-2 detection in clinical throat swab samples should be investigated.

5. I recommend authors to go through another round of editing prior to submission of the revision.

***1. Figure 1 mentioned the “Hammerhead Ribozyme”, but there is no introduction to the “Hammerhead”.***

We agree with the reviewer and have added the following text in the Introduction section to define and describe the structure of the Hammerhead Ribozyme (lines 70-73):

*Hammerhead ribozymes are small RNAs (~50–150 nt) capable of catalyzing their own self-cleaving and sequence-specific endonucleolytic cleavage. In solution, these ribozymes adopts a ‘Y’-shaped fold, with a secondary structure consisting of three double helices (helix I to III) intersecting in a three-way junction that contains the catalytic core of 15 highly conserved nucleotides (10).*

*Reference 10 (Garcia-Robles, 2012) was included.*

***2. A comparison with existing diagnostic methods such as RT-qPCR is suggested to highlight the advantages of the proposed method in terms of sensitivity, specificity, and cost-effectiveness.***

We agree with the reviewer's comment, and have added the following text at the begging of the Discussion section (line 677):

*Diagnostic methods based on nucleic acid amplification reactions, such as polymerase chain reaction (PCR), quantitative reverse transcription polymerase chain reaction (RT-qPCR), loop-mediated isothermal amplification (LAMP), helicase-dependent amplification (HDA), strand displacement amplification (SDA), and rolling circle amplification (RCA), are highly specific and sensitive. Several of these methods are used as viral diagnostic tests. However, other methodologies, such as those based on hybridization chain reaction (HCR), are potential cost-effectiveness alternatives compared to conventional thermal amplification reactions of nucleic acid. HCR can be performed under less parameterized conditions, without the need for more complex thermal cycling processes and the use of high precision instruments, thus reducing experimental costs (38-41).*

*Additionally, HCR exhibits high sensitivity and specificity, with the capability to recognize single-base mismatched DNA (42, 43). Moreover, the simplicity of HCR allows it to be integrated into various diagnostic platforms, including point-of-care devices, making it a versatile tool in molecular diagnostics. The ability to be conducted at a constant temperature further enhances its application in regions with limited sources, where traditional thermal cycling equipment is not feasible (44).*

New references have been included and updated.

***3. Some figures require improvement in clarity and labeling to ensure accurate conveyance of information.***

Thank you for your valuable feedback regarding the schematic figures. In response, we have revised the figures to ensure they meet the journal's standards and comply with the guidelines for authors. The updated figures are now formatted as high-quality .TIFF files with LZW compression. They are in RGB color mode (8 bit/channel), with dimensions ranging from 789 to 2250 pixels in width (at 300 dpi) and a maximum height of 2625 pixels (at 300 dpi). The resolution of the figures are 600 dpi. All text within the figures is set in Arial font, ranging from 8 to 12 points in size. Additionally, multi-panel figures have been combined into a single file for clarity and ease of review.

***4. SARS-CoV-2 detection in clinical throat swab samples should be investigated.***

We agree with the reviewer's comment. In principle, the implemented molecular methodology should work in various scenarios involving biological samples, especially throat swabs. The effectiveness of the diagnostic method will depend, among other factors, on the viral load or concentration of target RNA in biological samples. However, in the submitted manuscript, we only present the development of the methodology and it’s *in vitro* proof of concept using standard molecules. The use of the SARS-CoV-2 virus was mainly a circumstantial choice to demonstrate the functionality of the molecular method, rather than to develop a diagnostic procedure. However, based on the methodology we implemented, an appropriate diagnostic procedure could be developed. This would involve multiple validation, regulatory, and standardization studies, which would certainly include the use of throat swab samples.

***5. I recommend authors to go through another round of editing prior to submission of the revision.***

We would like to thank the reviewers for their comments and suggestions that were essential for improving the manuscript. As indicated by the reviewer, we conducted another round of review and editing based on reviewers´ concerns and suggestions. We believe that the majority of reviewers´ comments have been addressed and corrected, and that the article is now in better condition for publication.

Other minor corrections and modifications in the manuscript:

1. Line 365-366:

For the HCR-FRET assay three different final concentrations of the 18 nt initiator DNA molecule …

by

For the HCR-FRET assay, three different concentrations of the 18 nt initiator DNA molecule ….

2. Legenda Figure 3: Line 404-405:

The average fluorescence spectra emission (n = three independent experiments) were measured using the Varioskan …

by

Fluorescence emission spectra (n = three independent experiments) were recorded using the Varioskan …..

3. Legenda Figure 7: Line 507-509:

… while a single cleave by ribozyme 3 generates the segments of 121 nt and 279 nt (C). HCR-FRET may occur on 245 nt and 279 nt segments by the first DNA hairpins H1/H2 set.

by

… while a single cleavage by ribozyme 3 generates the segments of 121 nt and 279 nt (C). HCR-FRET may occur in segments of 245 nt and 279 nt by the first set of H1/H2 DNA hairpins.

4. Line 595:

… diagnostic process, relative to others methods, by ….

by

… diagnostic process, relative to other methods, by ….

5. Legenda Figure 11: Line 650:

The readings was carried out using the SpectraMax ….

by

The readings were carried out using the SpectraMax ….
